# Chemoenzymatic synthesis of the pH responsive surfactant octyl β-D-glucopyranoside uronic acid

**DOI:** 10.1007/s00253-019-10254-x

**Published:** 2019-12-07

**Authors:** Ngoc T. N. Ngo, Carl Grey, Patrick Adlercreutz

**Affiliations:** Division. of Biotechnology, Lund University, P.O. Box 124, 221 00 Lund, Sweden

**Keywords:** Oxidation, Octyl β-D-glucopyranoside, Octyl β-D-glucopyranoside uronic acid, Laccase from *Trametes versicolor*, TEMPO and foam

## Abstract

Methodology was developed to expand the range of benign alkyl glycoside surfactants to include also anionic types. This was demonstrated possible through conversion of the glycoside to its carboxyl derivative. Specifically, octyl β-D-glucopyranoside (OG) was oxidised to the corresponding uronic acid (octyl β-D-glucopyranoside uronic acid, OG-COOH) using the catalyst system *T. versicolor* laccase/2,2,6,6-tetramethylpiperidinyloxy (TEMPO) and oxygen from air as oxidant. The effects of oxygen supply methodology, concentrations of laccase, TEMPO and OG as well as reaction temperature were evaluated. At 10 mM substrate concentration, the substrate was almost quantitatively converted into product, and even at a substrate concentration of 60 mM, 85% conversion was reached within 24 h. The surfactant properties of OG-COOH were markedly dependent on pH. Foaming was only observed at low pH, while no foam was formed at pH values above 5.0. Thus, OG-COOH can be an attractive low-foaming surfactant, for example for cleaning applications and emulsification, in a wide pH range (pH 1.5–10.0).

**Electronic supplementary material:**

The online version of this article (10.1007/s00253-019-10254-x) contains supplementary material, which is available to authorized users.

## Introduction

Surfactants are used in a wide range of applications, but historically, they have caused severe environmental problems due to poor biodegradability, toxicity, and other undesirable properties. Alkylphenol-based surfactants are still used although they are known to be endocrine disruptors. On the other hand, alkyl glycosides constitute a group of benign surfactants with attractive functional properties (Hill 2010; von Rybinsky and Hill 1998). They can be produced from renewable raw materials and they are nontoxic and biodegradable. The range of available alkyl glycosides has been expanded by the extension of the carbohydrate head group by reactions catalysed by cyclodextrin glucanotransferase (Svensson et al. 2009). These oligomeric alkyl glycosides are particularly mild to living cells (Ulvenlund et al. 2016).

So far, mainly non-ionic alkyl glycosides have been studied. However, the introduction of ionisable groups can provide additional advantages, such as the possibility to modulate the surfactant properties by variation of pH. This has been studied in the case of acidic sophorolipids, surfactants which naturally contain carboxyl groups. Depending on pH, these lipids can form micelles, bilayers, vesicles or fibres (Baccile et al. 2016), while a yeast-derived glycolipid formed chiral nanoribbons at pH below 7.5 (Cuvier et al. 2014). In the present study, the introduction of carboxyl groups into nonionic alkyl glycosides was studied, using octyl-β-D-glucopyranoside (OG) as model substrate. The approach chosen was selective oxidation of the primary hydroxyl group of the substrate using 2,2,6,6-tetramethylpiperidinyloxy (TEMPO) as oxidant. Most commonly, TEMPO is used in catalytic amounts with NaOCl as stoichiometric oxidant (Bragd et al. 2004). The method has been used extensively for polysaccharide oxidation, giving products used for emulsion stabilization, thickening, controlled delivery systems, hydrogels, microspheres, etc. (Pierre et al. 2017).

To reduce the environmental impact of the TEMPO oxidation, oxygen can be used as the stoichiometric oxidant with a laccase as second catalyst (Bragd et al. 2004). Laccases are copper-containing enzymes, which have been widely used for selective oxidation reactions, and the most frequently used one for TEMPO oxidations is the one originating from *Trametes versicolor* (Larson et al. 2013). The traditional TEMPO oxidation method involving KBr and NaOCl has been used to oxidise alkyl glucosides and alkyl galactosides to the corresponding uronic acids (Milkereit et al. 2004). Furthermore, methyl glycosides have been oxidised to the corresponding uronic acids using electrochemical regeneration of TEMPO (Schamann and Schafer 2003) or using iodobenzene diacetate as oxidant (Lu et al. 2016). In a first attempt to adapt the green TEMPO/laccase system to alkyl glycoside oxidation, the oxidation of octyl-β-D-glucopyranoside was thoroughly studied. Of particular interest was to study if the methodology can be also applied to alkyl glycosides in micellar form, since most of the substrates of interest have critical micelle concentrations (CMC) in the μM to mM range.

## Materials and methods

### Materials

Laccase from *Trametes versicolor* as a powder, TEMPO (2,2,6,6-tetramethylpiperidine-1-oxyl), syringaldazine (4-hydroxy-3,5-dimethoxy benzaldehyde azine), citric acid and sodium citrate were purchased from Sigma-Aldrich (Sweden). Octyl-β-glucoside was obtained from Anatrace Inc., Maumee, Ohio, USA. All the chemicals were of analytical grade. Acetic acid, hydrochloric acid and sodium hydroxide were from Merck (Germany). Acetonitrile and propanol-2 were purchased from VWR (Sweden).

### Laccase activity assay

The laccase activity was determined spectrophotometrically by measuring the absorbance from the oxidation of syringaldazine at 530 nm at room temperature (Ride 1980). An assay solution which was composed of 0.88 ml of 0.1 M citrate buffer at pH 5, 0.2 ml of 0.1 mg/ml enzyme solution and 0.12 ml of 216 μM syringaldazine in methanol were added to a 1-ml cuvette. The enzymatic activity unit was defined as the amount of laccase converting 1 μmol of syringaldazine per minute.

### Laccase/TEMPO oxidation of OG

The oxidation reaction of OG was performed in triplicate in open glass vials under aeration. OG (typically 20 mM) and TEMPO (typically 19.2 mM) were dissolved in 3 ml of 100 mM citrate buffer at pH 5, followed by addition of laccase. The solution was shaken vigorously at 750 rpm at 24 °C. Samples (8 μl) were collected at scheduled times, diluted with DMSO (92 μl) and then analysed by HPLC-CAD and HPLC-MS. Initial reaction rate and conversion were quantified in terms of substrate (OG) consumption.

Similar experiments to investigate the effect of factors on the reaction rate and substrate conversion were performed with a range of TEMPO concentrations (6.4 to 36.3 mM), enzyme concentrations (17.6 to 212.5 U/l) and OG concentrations (10 to 60 mM). Besides, the influence of temperature ranging from 24 to 40 °C was studied. The investigation of different effects to supply oxygen was carried out by bubbling pure oxygen and shaking in normal atmosphere at 750 rpm.

### Analytical methods

HPLC-CAD analysis was performed using an UHPLC (Ultimate-3000 RSLC, Dionex) connected to a Charged Aerosol Detector (CAD) (Corona Veo), which was operated at evaporation temperature of 35 °C, and nebuliser gas pressure of 60.9 psi. A total of 2.5 μl of diluted sample was loaded onto a Kinetex Polar C18 column (Phenomenex, 100 Å, 2.6 μm, 2.1 × 150 mm) and eluted with acetonitrile (A): 0.1% acetic acid in water (B): 2-propanol (C) with a flow rate of 0.3 ml/min. The autosampler and column were set to 40 °C while the post column cooler was held at 30 °C. Gradient elution was carried out as follows: A/B/C held at 2:70:28 for 10 min, then changed to 2:50:48 within 2 min, held at this ratio for 2 min before returning to the initial conditions. Standard curves were used for quantification of OG and OG-COOH. No pure standard of OG-CHO was available, so in order to get approximate quantification of that product, the standard curve of OG-COOH was used, assuming equal response on weight basis, which is usually a good approximation when a CAD detector is used for quantification of similar substances.

The oxidation products of OG were identified by analysing mass spectra which were recorded on a HPLC-MS system. The HPLC (Thermo Scientific Accela) was connected to an Orbitrap Mass spectrometer composed of an electrospray ionization source (HESI-II) in negative mode with ion source voltage of − 2.5 kV, connected to a Velos Pro–Orbitrap mass spectrometer (Thermo Scientific, Waltham, MA, USA). Parameters for measurement were followed as source heater temperature 300 °C, capillary temperature 380 °C, sheath gas flow 35 AU, auxiliary gas flow 15 AU, sweep gas flow 1 AU. The column and elution method were the same as in the HPLC-CAD analysis.

### Product isolation and characterization

Isolation of octyl glucuronide was carried out by flash chromatography on silica gel RP18 (Spherical C18 bonded flash silica, Supelco Inc. USA). After the reaction finished, reaction mixture was heated to 100 °C for 15 min to inactivate the enzyme, and then freeze-dried before being loaded onto the chromatography column with a solvent system of 20% of methanol and 80% of aqueous acetic acid (0.15%, *v*/*v*). Elution was performed with a stepwise increase of MeOH from 20 to 80%. Fractions from the elution with 60% MeOH were collected and freeze-dried to furnish white powder. The structure of the product was determined analysing ^1^H NMR and ^13^C NMR spectra which were recorded on a Bruker DRX400 spectrometer at 400.13 MHz and 100.61 MHz using D_2_O to dissolve samples.

### Determination of pK_a_

The pK_a_ value of oxidised OG (OG-COOH) was determined by titration at 21 °C using a pH meter. OG-COOH solution (10 mM, 20 ml) was prepared and titrated with 20 mM sodium hydroxide. The experiment was repeated 3 times. Calculation of pK_a_ value is based on an Eq. 1. The titration curve showed an equivalence point and a half-equivalence point, at which pH is equal to pK_a_.

1 $$ pH=p{K}_a+\mathit{\log}\frac{\left[{A}^{-}\right]}{\left[ HA\right]} $$

### Foam ability and foam stability

The Bartsch method (Bougueroua et al. 2016) was applied to study foam formation and foam stability at different OG-COOH concentrations and varying pH values at 21 °C. An aqueous solution (10 ml) of OG-COOH was poured into a 100-ml cylinder, and pH of the solutions was adjusted using aqueous HCl or NaOH solution. Then, to make foams, the cylinder was turned upside down 10 times during 20 s. The foam heights at 0 min (*H*_*0*_) and 5 min (*H*_*5*_) after foam formation were measured to calculate the *R*_*5*_ parameter which was used to quantify the foam stability. Each experiment was repeated 2–3 times. The *R*_*5*_ value was calculated according to eq. 2.

2 $$ {R}_5=\frac{H_5}{H_0}\ast 100\% $$

## Results

**Scheme 1:**
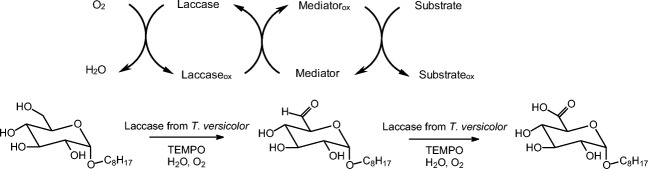
The general methodology to oxidise a substrate and octyl glucoside in particular using the O_2_/laccase/TEMPO system

The process (Scheme 1) studied includes the laccase-catalysed oxidation of a mediator with oxygen as oxidant and the chemical reaction between the oxidised mediator and the substrate OG. The combination of laccase from *T. versicolor* and the mediator TEMPO was chosen, based on reports from the literature comparing different enzymes and mediators in other oxidation reactions (Larson et al. 2013; Arends et al. 2006a; Mathew and Adlercreutz 2009). The kinetics of the overall process can be expected to be quite complex, because both an enzymatic conversion and a chemical step are involved. In this report, the factors—oxygen, TEMPO, enzyme, substrate and reaction temperature —on reaction rate and conversion of substrate were studied.

### Product isolation and characterization

The main reaction product isolated by flash chromatography was a white crystalline powder. The purity of product was estimated to be about 99% by NMR. By titration, pK_a_ was determined to be 3.32, slightly higher than that of β-D-glucuronic acid (pK_a_ = 2.83) (Wang et al. 1991). Mass spectrometry and NMR spectroscopy confirmed that the product was octyl β-D-glucopyranoside uronic acid. HR-ESI-MS [M-H]^−1^: calculated for C_14_H_25_O_7_: m/z 305.1606. Found: 305.1605. ^1^H NMR (400 MHz, D_2_O): *δ*_*H*_ 4.44 (1H, d, 9.8 Hz, H-1), 3.93 (1 H, d, 12.0 Hz, H-5), 3.85 (1 H, dt, 11.7 and 9.1 Hz, H-1a′), 3.60 (2 H, m, H-1b′, H-4), 3.52 (1 H, t, 11.5 Hz, H-3), 3.35 (1 H, dd, 11.4 and 10.0, H-2), 1.61 (2H, quint, 8.5 Hz, H-2′), 1.23 (10H, m, H-3′ to H-7′), 0.83 (3H, t, 7.0 Hz, H-8′). ^13^C NMR (100 MHz, D_2_O): *δ*_*C*_ 172.0 (C-6), 102.4 (C-1), 75.3 (C-3), 74.7 (C-5), 72.7 (C-2), 71.3 (C-4), 70.6 (C-1′), 31.6, 29.1, 25.5, 22.4 (C-2′ to C-7′), 13.6 (C-8′). Data agree with previous reports (Ferlin et al. 2008).

An intermediate product was formed during the initial part of the reaction that was further converted to OG-COOH. This was identified as the corresponding aldehyde (OG-CHO). HRESIMS [M-C_2_H_4_O_2_]^−1^: calculated for C_14_H_25_O_6_: m/z 349.1868. Found: 349.1876.

### Oxygen supply

The oxidation reaction mixture composed of OG 20 mM, TEMPO 19.2 mM and laccase 70.8 U/l was prepared to study the influence of different ways to supply oxygen. When passing a stream of pure oxygen continuously through the reaction mixture at 24 °C, the dissolved oxygen concentration can be assumed to be 1.30 mM (Tromans 1998) and the observed initial reaction rate was 26.0 μM/min. When the reaction mixture was just shaken in normal atmosphere, the initial reaction rate was 23.0 μM/min. Under those conditions, the dissolved oxygen concentration can be assumed to be 0.27 mM, which was apparently high enough to keep the laccase rather close to saturation with respect to oxygen. This observation agrees with reported values of *K*_M_ values of *T. versicolor* laccase for oxygen in the range of 20–50 μM (Xu 2001). After longer reaction time, higher conversion was observed in the case of shaking in air (90.38 ± 0.09%) than with oxygen bubbling (86.04 ± 0.97%). This might be due to inactivation of the laccase at the gas-liquid interface formed by the oxygen bubbles. In previous studies of laccase-catalysed oxidation, oxygen supply from air and pure oxygen has given similar reaction rates (Arends et al. 2006b). Because of the possibilities to reach higher final conversions and the more practical experimental set-up, oxygenation by shaking in air was chosen for the rest of the study.

### TEMPO concentration

**Fig. 1:**
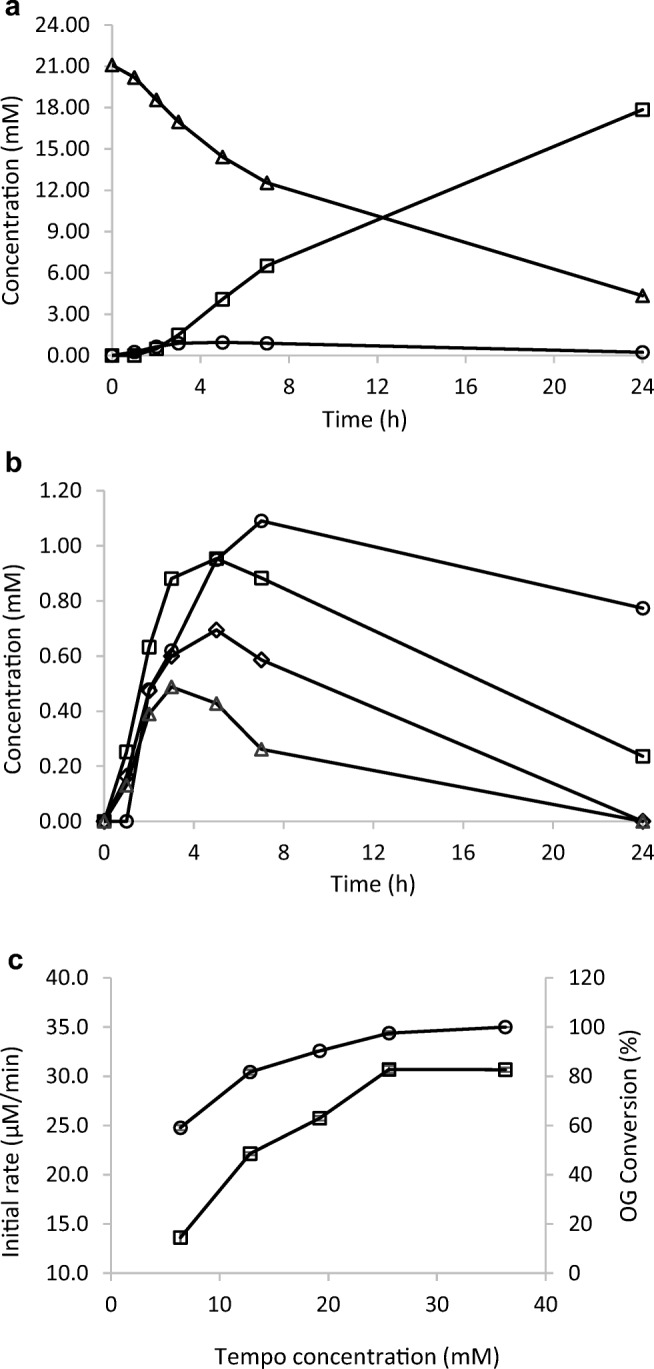
Influence of TEMPO concentration on the OG oxidation. **a** The time course of consumption of OG (∆) and formation of oxidation products: OG-CHO (○) and OG-COOH (□). Reaction conditions: OG (20 mM), TEMPO (12.8 mM), laccase (70.8 U/l). The oxidation was carried out at 24 °C with shaking at 750 rpm. **b** The time course of the formation of aldehyde (OG-CHO) at varying TEMPO concentrations. Reaction conditions: OG (20 mM), laccase (70.8 U/l) and different TEMPO concentrations (○ 6.4 mM, □ 12.8 mM, ◊ 25.6 mM, ∆ 36.3 mM) at 24 °C with shaking at 750 rpm. **c** Influence of TEMPO concentration on the initial rate (□) and conversion (○) from substrate consumption after 24 h. Reaction conditions: OG (20 mM), laccase (70.8 U/l) and different TEMPO concentrations at 24 °C with shaking at 750 rpm

In a similar way as in previously reported TEMPO-laccase oxidation reactions (Arends et al. 2006a; Mathew and Adlercreutz 2009), the primary alcohol of OG was oxidised to an aldehyde (named OG-CHO), which was subsequently converted to an acid (OG-COOH). As shown in Fig. 1a, the amount of OG-CHO formed was much smaller than OG-COOH, because of rapid conversion of OG-CHO to OG-COOH. In this secondary oxidation reaction, both oxygen and TEMPO seem to have been involved (Jausovec et al. 2015; Li et al. 2013). The fact that the amount of aldehyde observed decreased with increasing TEMPO concentration shows that TEMPO promoted aldehyde oxidation (Fig. 1b) (Marjasvaara et al. 2004) and a high TEMPO concentration thus favours full conversion to OG-COOH.

TEMPO is the substrate which is directly oxidised by laccase. In previous reports on oxidation reactions involving *T. versicolor* laccase, a wide range of TEMPO concentrations have been used. Most commonly, the TEMPO concentration has been in the range 4–30 mM (Larson et al. 2013; Arends et al. 2006b; Marjasvaara et al. 2004), but even concentrations as low as 0.1 mM have been used (Jausovec et al. 2015). In the present study, the influence of the TEMPO concentration on the rate of OG concentration appeared to obey Michaelis Menten kinetics (Fig. 1c) with a K_M,app_ value of 12.8 mM. This is in the same order of magnitude as the *K*_M_ value of 6.3 mM reported for the same enzyme and 4-acetylamino-TEMPO in the oxidation of furfuryl alcohol (Arends et al. 2006b). A TEMPO concentration of 19.2 mM was chosen for the rest of the study.

### Enzyme concentration

In purely enzymatic reactions, the initial reaction rate is expected to be proportional to the enzyme concentration unless mass transfer limitations are significant. However, in the case studied, the chemical reaction step makes the situation more complex, so a careful study of the effect of the enzyme concentration was motivated. The reactions were performed with fixed TEMPO concentration of 19.2 mM and various enzyme concentrations from 17.6 to 212.5 U/l. At low enzyme concentrations (< 42.5 U/l), the reaction rate was almost proportional to the enzyme concentration, but at higher enzyme concentration, the curve flattened out (Fig. S1). These results indicated that 141.7 U/l was a sufficient enzyme concentration to get high yield.

### OG concentration

**Fig. 2:**
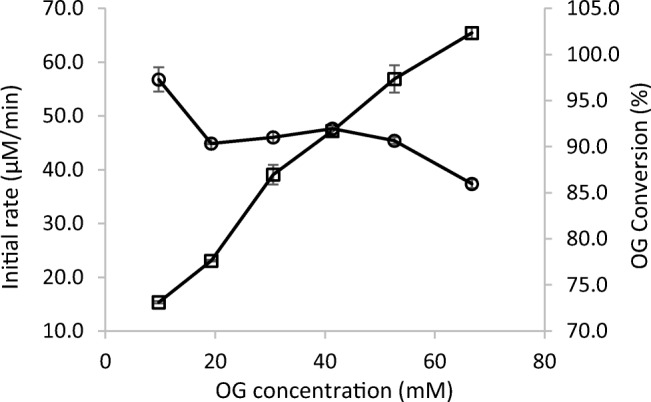
Effect of OG concentration on the initial reaction rate (□) and conversion from substrate consumption (○) after 24 h. Reaction conditions: TEMPO (19.2 mM), laccase (70.8 U/l) and different OG concentrations, 24 °C, shaking at 750 rpm

Oxidised TEMPO reacts with OG in a chemical reaction. The overall initial reaction rate increased with increasing OG concentration. The results agree with the previous observation of pseudo first-order kinetics in the oxidation of furfuryl alcohol in a similar system (Arends et al. 2006b). It should be noted that OG is a surfactant and thus to a large extent exists as micelles at concentrations above the critical micelle concentration, which in the case of OG is about 22 mM (Bergeron et al. 1996). It seems that the oxidation process worked well not only with monomeric but also with micellar substrate (Fig. 2), which is important especially if the method is to be applied to surfactants having even lower critical micelle concentrations. A conversion of at least 85% was achieved after 24 h, even at a substrate concentration exceeding 60 mM.

### Reaction temperature

**Fig. 3:**
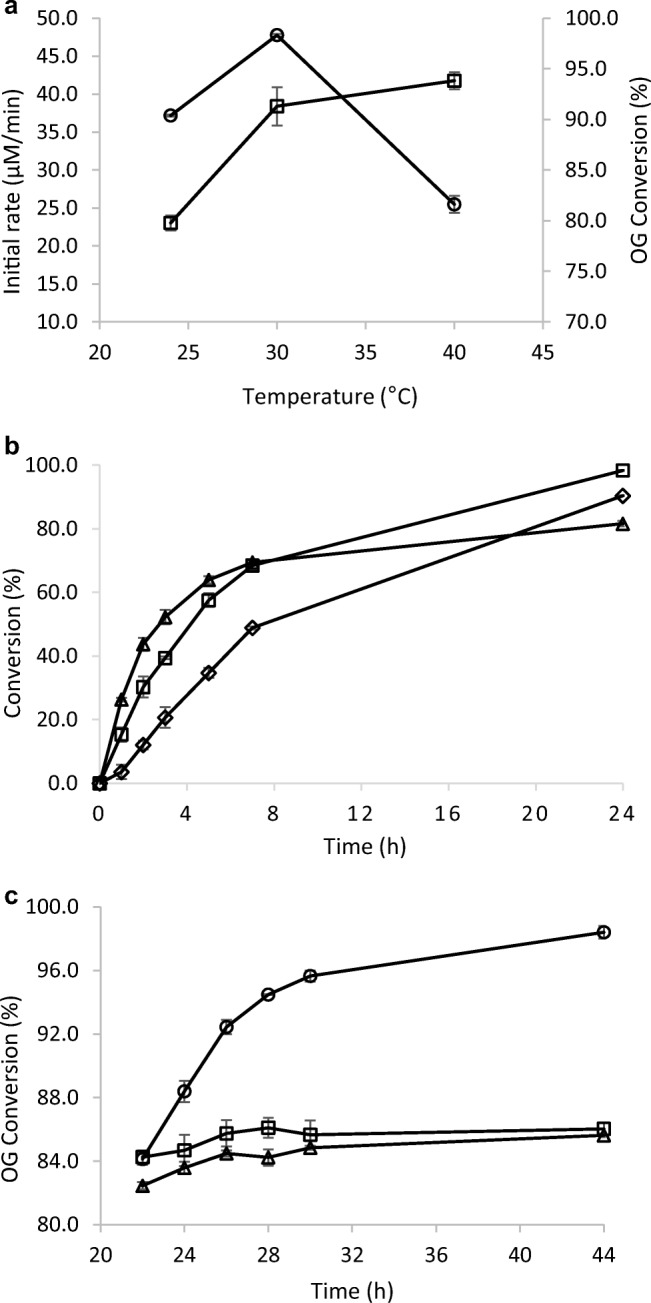
Effect of temperature on the OG oxidation. Reaction conditions: OG (20 mM), TEMPO (19.2 mM) and laccase (70.8 U/l) at 24 °C, 30 °C and 40 °C, shaking at 750 rpm. **a** Influence of temperature on the initial rate (□) and conversion (○) from substrate consumption after 24 h. **b** The time course of the conversion of OG at three different temperatures (◊ 24 °C, □ 30 °C and ∆ 40 °C). **c** Reactivation of the laccase/TEMPO oxidation system. The oxidation reaction was carried out at 40 °C for 22 h. After 22 h, the reaction had virtually stopped and attempts to restart the conversion were made by addition of TEMPO (19.2 mM) (∆), laccase (70.8 U/l) (○) or nothing (□) and the time course of the three reactions was recorded

The initial reaction rate increased considerably with increasing temperature between 24 and 40 °C in spite of the poor solubility of oxygen in water at elevated temperature (Fig. 3a). However, when the reaction was prolonged, at 40 °C, after 7 h, the reaction rate decreased, while at 30 °C close to full conversion was achieved after 24 h (Fig. 3b). In order to find out why the reaction virtually stopped after 24 h at 40 °C, the effects of extra additions of either laccase or TEMPO were made to the reaction mixtures. The addition of more laccase caused the reaction to continue to close to quantitative conversion, which indicates that enzyme inactivation was the limiting factor (Fig. 3c). The addition of extra TEMPO had only minor effects, indicating that TEMPO degradation was not the prime cause of slowing down the reaction. The above results indicate that laccase expressed the highest activity at 40 °C but was degraded at extended reaction time, while good stability was observed at 30 °C.

### Foamability and foam stability

The ability to cause foaming is an important property of surfactants. In some applications, foaming is desired, for example, in shampoos and other personal care products, while in other applications, foaming constitutes a problem. It can, for example, be of interest to use surfactants to create emulsions or for various cleaning applications, and then, foam formation is often not desired. In the case of OG-COOH, there is a possibility that its surfactant properties can be influenced by adjusting pH. To make a first study of these possibilities, foaming was studied in a range of pH values from 1.6 to 10. CMC of OG-COOH has been reported to be 15.6 mM (Ferlin et al. 2008), and in order to evaluate its foaming properties both below and above CMC, the surfactant concentrations 10 and 20 mM were chosen.

**Fig. 4:**
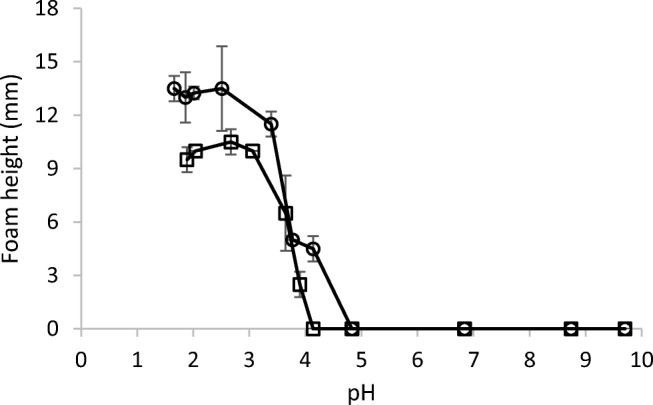
Plot of the initial foam height as function of pH value for OG-COOH concentrations of 10 mM (□) and 20 mM (○)

Efficient foam formation occurred at low pH values, while foam height decreased significantly around the pK_a_ value of OG-COOH and at pH values of 5 and above, no foam was observed (Fig. 4). Foam formation occurred both below and above CMC, with more foam at the higher concentration.

In addition to the initial foam volume, the stability of the foam is of interest. Foam stability was estimated by calculating the *R*_*5*_ value, the relative amount of foam remaining 5 min after its formation. Foam stability decreased with increasing pH, and at pH values of 3.8 and above, no foam at all remained after 5 min (*R*_*5*_ = 0) (Table S1).

## Discussion

Selective oxidation of hydroxyl groups in carbohydrate derivatives is usually hard to accomplish. Here, the production of OG-COOH from OG was achieved using the TEMPO/laccase/O_2_ system in water at room temperature. Compared with chemical methods, using protection and deprotection of other hydroxyl groups (Ferlin et al. 2008) or chemoselective catalysts (Boelrijk et al. 1996), the laccase/TEMPO mediated oxidation was shown to be a promising green alternative.

Generally, this is a chemoenzymatic reaction in which TEMPO is oxidised by laccase to oxoammonium TEMPO, which in turn oxidises the primary alcohol of OG to a carbonyl group, and subsequently to a carboxyl group. Therefore, both TEMPO and laccase concentrations significantly affected the reaction rate. Furthermore, it has been reported that the oxoammonium TEMPO acts inactivating on the laccase and that this effect is reduced by the presence of an alcohol substrate to be oxidised (Arends et al. 2006b; Jiang et al. 2017). This means that the increased reaction rate caused by increased TEMPO concentration comes with the price of lower enzyme stability. It should be pointed out that the oxidation of OG continued to full conversion at the highest TEMPO concentration (Fig. 1c), thus indicating that the extent of enzyme inactivation was moderate under the conditions used. Furthermore, when increasing the enzyme concentration from 141.7 to 212.5 U/l, the initial reaction rate did not increase as expected. This could be due to, once again, the inactivation of laccase by oxoammonium TEMPO which could be formed to a considerable extent at a high enzyme concentration, or to nonenzymatic step(s) beginning to limit the overall reaction rate, or to limitation of oxygen which was employed as a first oxidant. The possibility that the reaction product OG-COOH could inactivate the laccase was investigated experimentally and no inactivation was noted (results not shown). This is also in good agreement with a previous study of laccase stability with the presence of surfactants (Azimi et al. 2016). In general, it is encouraging that a conversion of 85% was reached, even at a substrate concentration exceeding 60 mM.

OG is surface active and is capable of forming stable foams from pH 6 to 10, but has very poor foam capacity at pH 5 and below (Bergeron et al. 1996). Meanwhile, foaming of oxidised OG, OG-COOH, decreased with increasing pH and no foam was observed at pH above 5. It is thus obvious that the difference in pH dependence of foaming between these two compounds is due to the introduction of the carboxyl group. The most straightforward explanation of the pH dependence of foaming of OG-COOH is that the protonated form favours foam formation while the anionic form does not. The drop in the foam height correlates well with the measured value of pK_a_ (3.32). At pH values above pK_a_, the surfactant is negatively charged causing electrostatic repulsion and an increase in hydration and thereby an increase in the area per head group (Micheau et al. 2013) producing less cohesive forces at the surface (Chen and Tsai 1988). Similarly, electrostatic repulsion was suggested as the explanation for decreased foam stabilization by bovine serum albumin above or below its isoelectric point (Engelhardt et al. 2012). Another factor of importance could be strong charge-dipole interactions between charged and uncharged surfactant molecules, which are maximal around pK_a_ when about equal amounts of the two forms are present. These forces have been claimed to cause maxima in foam formation of fatty acid soaps around their apparent pK_a_ values (Kanicky et al. 2000). It is worth noting that foaming was totally absent for OG-COOH at pH values > 5, while fatty acid salts caused some soap formation also at pH values far from the pH optima.

In conclusion, the production of OG-COOH by selective oxidation on the primary alcohol group of OG using laccase/TEMPO system was demonstrated. In spite of laccase being partially inactivated during the reaction, the system still worked well to consume virtually all the primary alcohol in the substrate and achieve good yields of the carboxylic acid product. Previously, alcohols, ethers, starch, and cellulose have been studied as substrates in this type of conversion. Here, it was applied for the first time on an alkyl glycoside surfactant. In this study, the reaction worked with OG as either free molecules or in micellar form to reach high conversion. It should thus be applicable also to alkyl glycosides with lower CMC values, such as alkyl glycosides with longer alkyl chains. A group of especially interesting substrates are the alkyl polyglucosides, which are produced in large amounts and widely used for practical applications. Their main components are alkyl glycosides having just one glucose residue and they should thus be good starting materials for conversion with the methodology presented. The fact that OG-COOH has very low tendency to form foam in a wide range of pH values (pH > 5) can make it very attractive for applications where foam formation is not desired. Furthermore, the possibility to modulate the extent of foam formation by a change in pH might be useful when a switch in foaming is needed. It should be pointed out that it is highly probable that other surface activities of OG-COOH also can be modulated by pH changes, so it can be considered as a promising pH responsive surfactant.

## Electronic supplementary material

ESM1 (PDF 147 kb)
