# Supplementary material for: Chemoenzymatic synthesis of the pH responsive surfactant octyl β-D-glucopyranoside uronic acid
Source: Appl Microbiol Biotechnol. 2019 Dec 7;104(3):1055–62. doi: 10.1007/s00253-019-10254-x (PMC6962123; doi:10.1007/s00253-019-10254-x)

Chemoenzymatic synthesis of the pH responsive surfactant octyl β-D-glucopyranoside uronic acid

Ngoc T. N. Ngo, Carl Grey and Patrick Adlercreutz
Div. of Biotechnology, Lund University, P.O. Box 124, 221 00 Lund, Sweden

Email of corresponding author: Patrick.adlercreutz@biotek.lu.se

Supporting information

Table

The foam heights at 0 min (H_0) and 5 mins (H_5) after OG-COOH foam formation were measured to calculate the R_5 parameter which was used to quantify the foam stability. The R_5 value was calculated according to equation 2.

$$R_5 = \frac{H_5}{H_0} * 100\% \qquad \text{(Eq. 2)}$$

Table S1. Residual foam height ratio R_5

**OG-COOH 10 mM**		**OG-COOH 20 mM**	
pH	R_5 (%)	pH	R_5 (%)
1.89	10.5	1.66	51.9
2.04	10.0	1.86	53.8
2.67	14.3	2.01	52.8
3.06	0.0	2.51	53.7
3.65	0.0	3.39	17.4
3.90	0.0	3.77	0.0

Figures

Figure S1. Effect of enzyme concentration on the initial rate of oxidation of OG (□) and conversion (○) from substrate consumption after 24 h. Reaction conditions: OG (20 mM), TEMPO (19.2 mM) and different enzyme concentrations, 24 °C, shaking at 750 rpm.

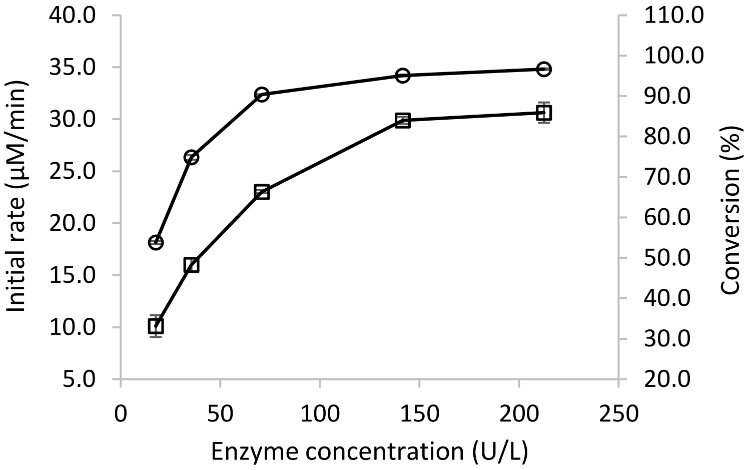

Supplement: Supplementary file 1 — (PDF 147 kb) [file 253_2019_10254_MOESM1_ESM.pdf]
